# Quality Improvement Project to Develop a Pictorial Chronic Obstructive Pulmonary Disease (COPD) Action Plan

**DOI:** 10.7759/cureus.68171

**Published:** 2024-08-30

**Authors:** Yiting Tang, QinHao Jonathan Ye, Hsiao Peng Toh, Jessica Tan, Gan Liang Tan, Kiran Sharma

**Affiliations:** 1 Respiratory Medicine, Sengkang General Hospital, Singapore, SGP; 2 Internal Medicine, Sengkang General Hospital, Singapore, SGP; 3 General Medicine, Sengkang General Hospital, Singapore, SGP

**Keywords:** copd self management, pictorial action plan, copd action plan, written action plan, copd (chronic obstructive pulmonary disease)

## Abstract

Introduction

Chronic obstructive pulmonary disease (COPD) is a global health concern and a leading cause of morbidity and mortality worldwide. COPD action plans help patients manage exacerbations by recognizing symptoms early and taking necessary steps. We found our COPD written action plan difficult to understand, potentially affecting the patient’s ability to self-manage their COPD.

Aims

We aim to design a new COPD action plan to increase the knowledge scores of our patients during competency checks by 20%.

Methods

We employed the quality improvement methodology of needs analysis and root cause analysis and used a Pareto chart to identify the top four contributory factors to an ineffective COPD action plan. These include being too wordy, lacking pictorial illustrations, being only available in a single language (English), and too much medical jargon. Using the prioritization matrix to assess possible solutions, the team decided to implement a pictorial COPD action plan. After two cycles of Plan-Do-Study-Act, the final pictorial COPD plan was compared with the original written action plan.

Results

Ten English-speaking COPD patients from our outpatient respiratory clinics were surveyed with the original action plan while 11 more were surveyed after the introduction of the pictorial action plan. There was an improvement in mean knowledge scores by 92.8% (t(19) = 6.67, p < 0.01, at 95% CI). Patient satisfaction rates also increased from 44% to 100%. Sixty-three percent (63.6%) of patients surveyed said they referred back to the pictorial action plan 3 months after being introduced to it.

Conclusion

Pictorially enhanced COPD action plans have been shown to improve our patients’ knowledge of COPD self-management.

## Introduction

Chronic obstructive pulmonary disease (COPD) is a global health concern and a leading cause of morbidity and mortality worldwide. COPD imparts a significant economic burden, including both direct costs of healthcare resource utilization and indirect costs of lost work productivity [1,2]. It is the tenth leading cause of death in Singapore [3].

COPD action plans help patients manage exacerbations through early recognition of symptoms and taking necessary steps, such as administering antibiotics and oral corticosteroids [4]. These plans have been proven effective in Cochrane reviews [5].

## Materials and methods

COPD action plan

In the 2022 Cochrane review of self-management interventions in patients with COPD, the COPD action plan was mentioned to be associated with improvements in health-related quality of life, as well as reductions in respiratory-associated admissions and emergency department (ED) admissions [6].

These self-management interventions frequently rely on the patient's capability to recognize an exacerbation and initiate the action plan. As a result, the success of any action plan-based strategy would be primarily reliant on the patient’s comprehension and ability to become an effective self-manager.

**Figure 1:**
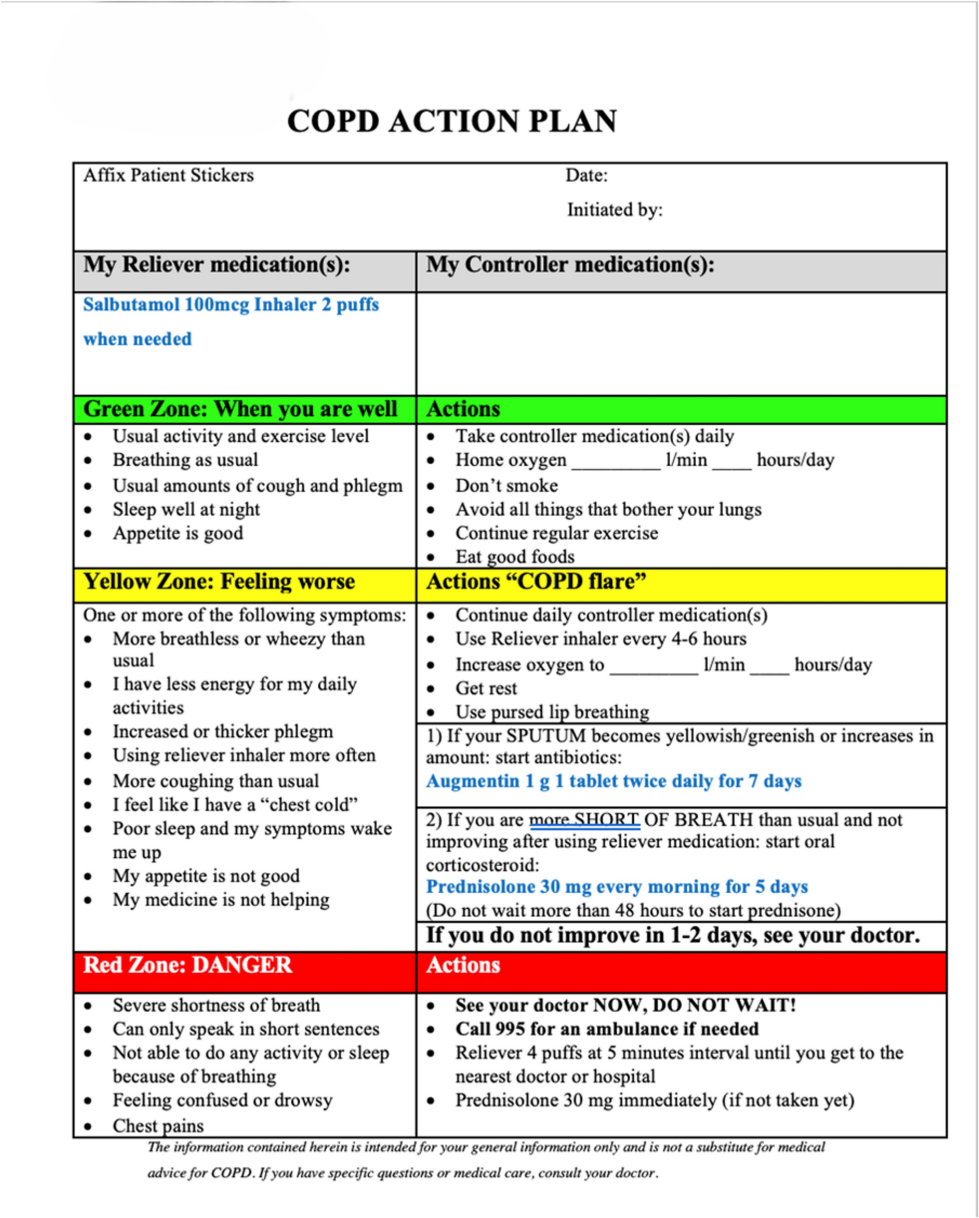
Sengkang General Hospital’s original COPD action plan Adapted from the American Lung Association and the Canadian Thoracic Society [7,8] COPD: chronic obstructive pulmonary disease

Our hospital has an established COPD action plan (Figure 1), which is adapted from both the American Lung Association [7] and the Canadian Thoracic Society [8]. These action plans are given out to our COPD patients by doctors and respiratory specialist nurses. We have received feedback from quite several patients and their caregivers that our current COPD action is difficult to understand. This could negatively impact our patient’s ability to manage exacerbations effectively, leading to poor clinical outcomes. Therefore, we decided to enhance our action plan to ensure that it is easy to comprehend [9].

Context

This quality improvement (QI) initiative was carried out at the Sengkang General Hospital Respiratory Medicine Department to improve our COPD action plan. A multidisciplinary QI team was established to investigate and carry out a QI project using the Institute for Healthcare Improvement's (IHI) Plan-Do-Study-Act (PDSA) framework. The framework involves a four-stage cyclic process for implementing and refining improvements. The team consisted of specialist nurses, pharmacists, senior residents, and consultants from the respiratory medicine department.

Needs analysis

To gain a better understanding of the attitudes and opinions of doctors, nurses, pharmacists, patients, and their caregivers regarding the current action plan, we conducted a needs analysis. This involved distributing questionnaires (Table 1) to all the relevant stakeholders. They consist of four questions meant to assess whether the respondents feel that the COPD action plan is important, if the current action plan is difficult to understand, if there is a need to improve it, and lastly, some of the factors that have contributed to an ineffective COPD action plan.

**Table 1:** Sample of a Needs Analysis questionnaire COPD: chronic obstructive pulmonary disease

		Strongly disagree	Disagree	Agree	Strongly Agree
1.	The COPD action plan is important to help improve self-management knowledge among our patients				
2.	The current COPD action plan is difficult to understand				
3.	There is a need to improve our current COPD action plan				
4.	What are some of the contributory factors to an ineffective COPD action plan?	­			

We collected a total of 40 responses (Table 2) from 9 patients and their caregivers, 14 doctors, 5 nurses, and 12 pharmacists. Our needs analysis showed that most of the respondents strongly agree (n=25, 62.5%) that the COPD action plan is important to help improve the self-management knowledge of patients. Sixty-two point five percent (62.5%) of responses agree or strongly agree that there is a need to improve it.

**Table 2:** Responses from the Needs Analysis questionnaire COPD: chronic obstructive pulmonary disease

Overall Response (n=40)							
		Strongly Disagree n (%)	Disagree n (%)	Agree n (%)	Strongly Agree n (%)		
1	The COPD action plan is important to help improve self-management knowledge among our patients	1 (2.5)	0 (0)	14 (35)	25 (62.5)		
						2	The current COPD action plan is difficult to understand	1 (2.5)	21 (52.5)	14 (35)	4 (10)		
3	There is a need to improve our current COPD action plan	2 (5)	13 (32.5)	19 (47.5)	6 (15)								
						Response from pharmacists, nurses, and doctors (n=31)							
								Strongly Disagree n (%)	Disagree n (%)	Agree n (%)	Strongly Agree n (%)		
1	The COPD action plan is important to help improve self-management knowledge among our patients	1 (3.2)	0 (0)	13 (41.9)	17 (54.8)		
						2	The current COPD action plan is difficult to understand	1 (3.2)	19 (61.3)	9 (29.0)	2 (6.5)		
3	There is a need to improve our current COPD action plan	2 (6.5)	11 (35.5)	15 (48.4)	3 (9.7)								
						Response from patients (n=9)							
								Strongly Disagree n (%)	Disagree n (%)	Agree n (%)	Strongly Agree n (%)		
1	The COPD action plan is important to help improve self-management knowledge among our patients	0 (0)	0 (0)	1 (11.1)	8 (88.9)		
						2	The current COPD action plan is difficult to understand	0 (0)	2 (22.2)	5 (55.6)	2 (22.2)		
3	There is a need to improve our current COPD action plan	0 (0)	2 (22.2)	4 (44.4)	3 (33.3)								

It is interesting to note that 52.5% of our respondents do not find our current action plan difficult to understand. However, when the data is analyzed according to the various subgroups, there is a difference of opinions between our patients and healthcare staff. The majority of the patients (n=7, 77.8%) agree or strongly agree that the action plan is difficult to understand while only a small proportion (n=11, 35.5%) of our healthcare professionals (doctors, nurses, pharmacists) felt the same way.

Assessment of problems

**Figure 2:**
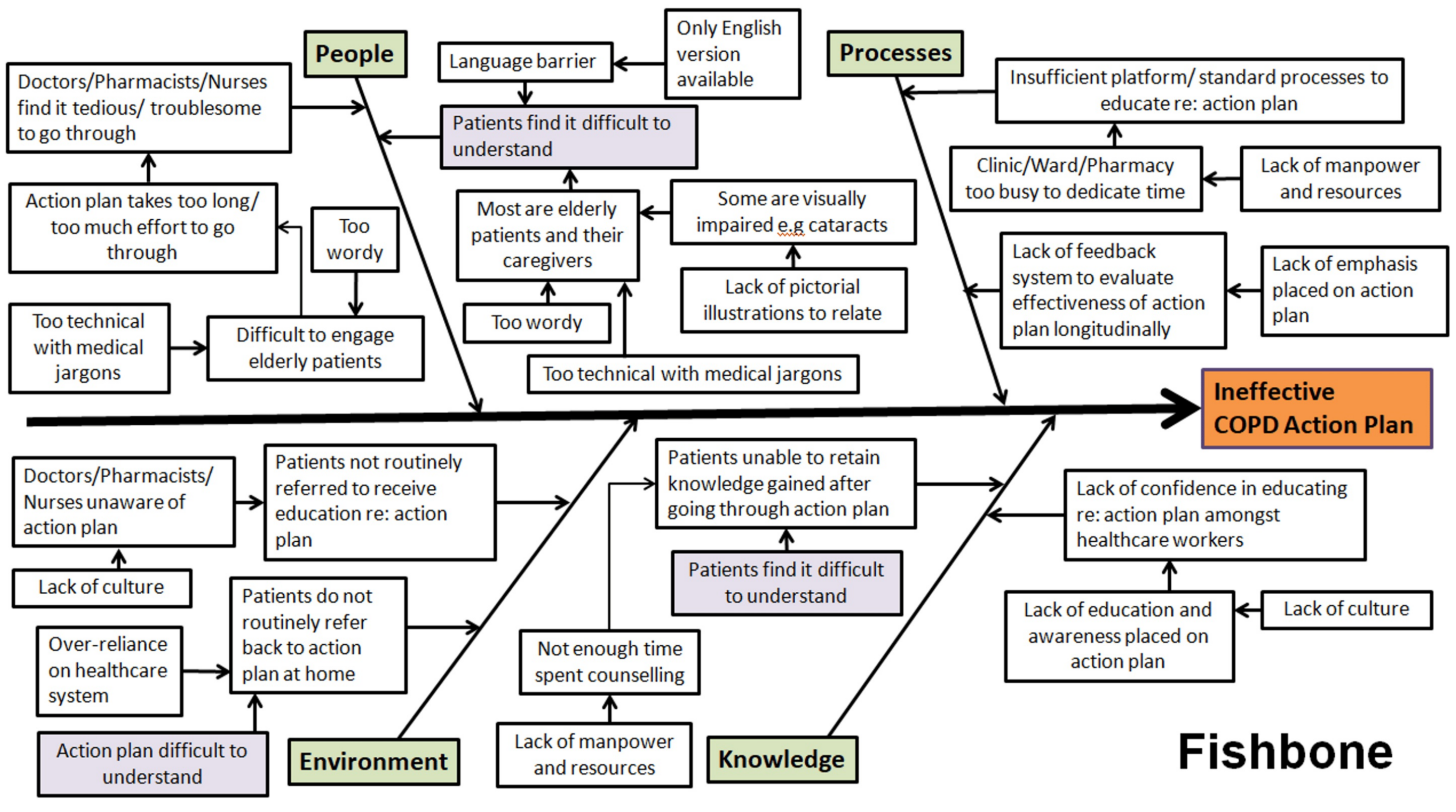
Fishbone cause-and-effect analysis Image credit: The authors COPD: chronic obstructive pulmonary disease

**Figure 3:**
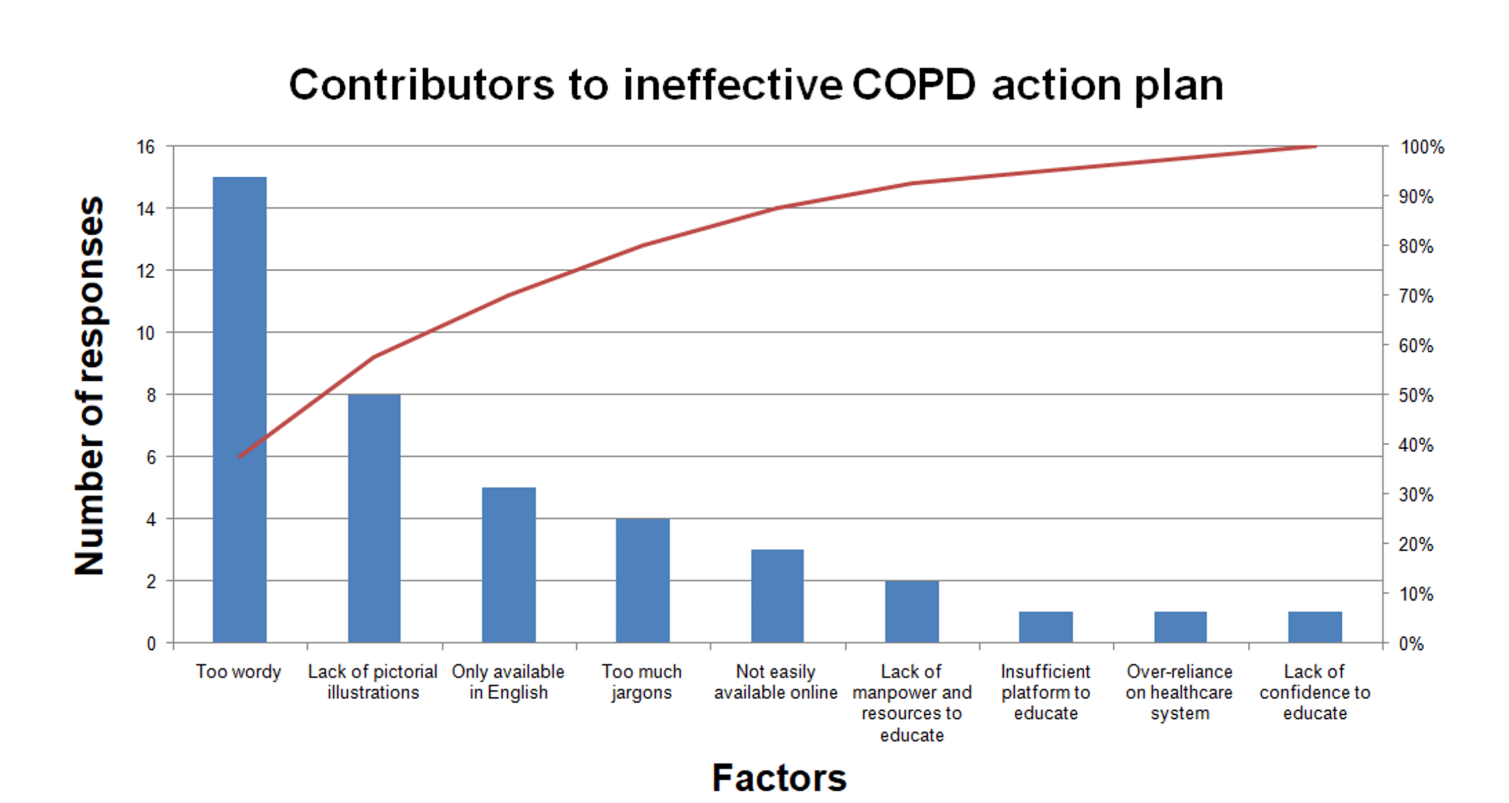
Pareto chart Image credit: The authors

A root cause analysis was first performed by the QI team to identify factors that contributed to an ineffective COPD action plan. A Fishbone diagram was constructed to list the People, Environment, Processes, and Knowledge factors (Figure 2). Next, the team ran a Pareto chart (Figure 3) using responses from our stakeholders to identify the most important contributory factors to an ineffective COPD action plan. The Pareto chart is based on the Pareto principle, which states that about 80% of the outcome comes from 20% of its inputs. Hence, taking 80% of the responses we obtained from the Needs Questionnaire as a cut-off, we managed to identify the top four contributory factors. These include being too wordy, lack of pictorial illustrations, availability only in a single language (English), and too much medical jargon.

After identifying the top four contributory factors, the QI team came together to brainstorm various intervention options. These ideas were placed in a Prioritization Matrix (Table 3) and assessed based on their reliability, effectiveness, feasibility, affordability, and sustainability. Ultimately, the option of having a pictorially augmented COPD action plan obtained the highest score in our assessment and therefore, was chosen to be our primary intervention for the project.

**Table 3:** Prioritization matrix

	Weight of each attribute (1 ~ 9)	6	7	8	3	6	30	
		12%	23%	27%	23%	20%	100%	100%
	Type of option (Project’s potential changes)	Reliability	Effective	Feasible	Low cost	Sustainable	Score	Score mark
Options	Pictorial action plan	8	8	9	5	9	8	||||||||
	Increase clinic consult time to educate the action plan	3	7	1	1	2	3	||
	Create QR code/digitalize action plan	5	5	8	3	5	6	|||||
	Translate to different languages	8	8	9	5	8	8	|||||||
	Interactive video action plan	5	5	6	3	4	5	||||

QI objective

A pictorially augmented COPD action plan was chosen to better engage our COPD patients. By designing and developing a new action plan, we aim to increase the knowledge scores of our patients during competency checks by 20% over a 10-month period.

Data collection and analysis

**Figure 4:**
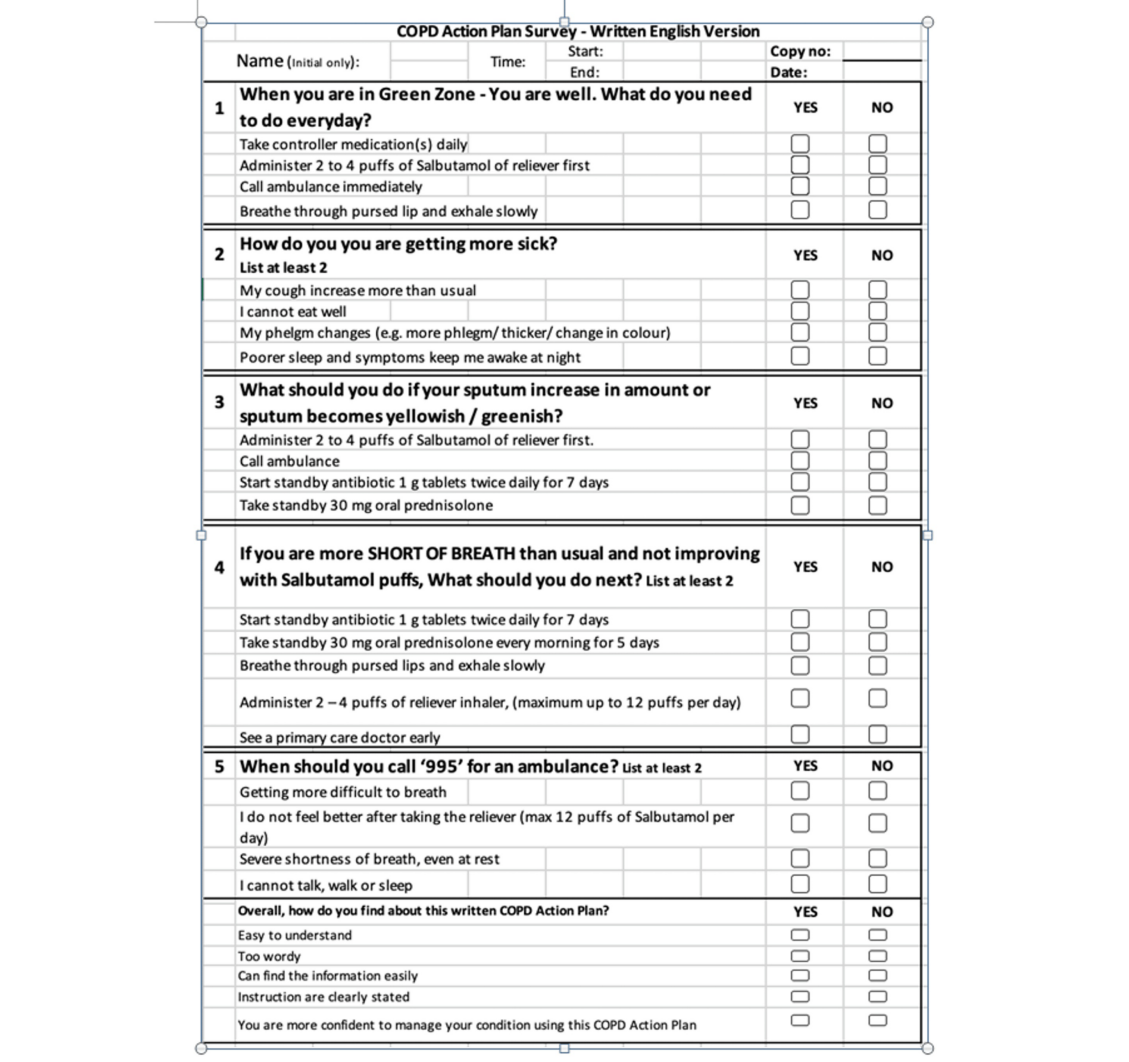
COPD action plan competency checklist Adapted from: [15] COPD: chronic obstructive pulmonary disease

Data collection was conducted at two different time frames, first using the existing COPD action plan, followed by using the newly developed COPD action plan. Both action plans were issued to our patients by the respiratory specialist nurse during outpatient clinic follow-ups. After going through the contents of the action plan, patients would undertake a five-question competency check (Figure 4) to assess their understanding of COPD self-management via structured questioning. We allocated 2 points for each question. Patients got 1 point if they managed to list one correct answer, and 2 points if they managed to list at least two correct answers and above. Knowledge scores were then derived after tabulating the total number of points obtained. At the end of the survey, patients were also asked about how satisfied they were with the action plan.

Three months after administration of the pictorial COPD action plan, our patients received a phone call from our QI team to check back on the utilization rates of the action plan.

The above data were de-identified and securely stored in a password-encrypted Microsoft Excel-based database (Microsoft Corporation, Redmond, WA, US). It was subsequently shared with the QI team for data analysis.

Plan-Do-Study-Act (PDSA)

A total of two PDSA cycles were carried out.

**Figure 5:**
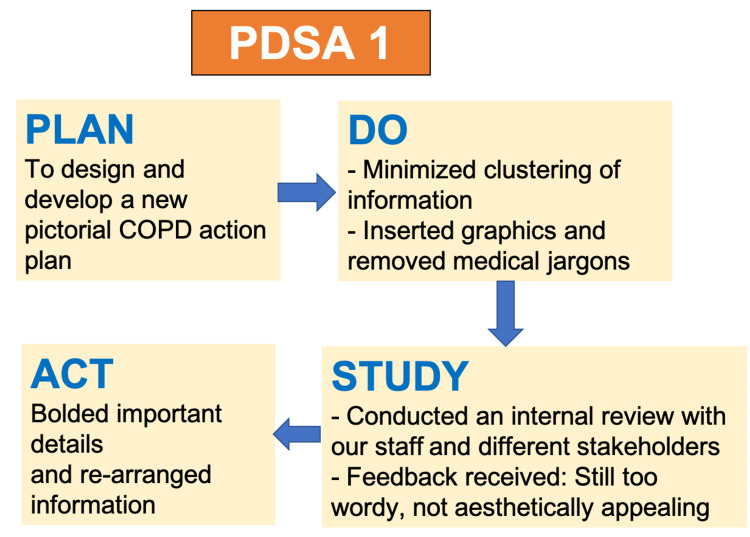
PDSA Cycle 1 Image credit: The authors PDSA: Plan-Do-Study-Act

**Figure 6:**
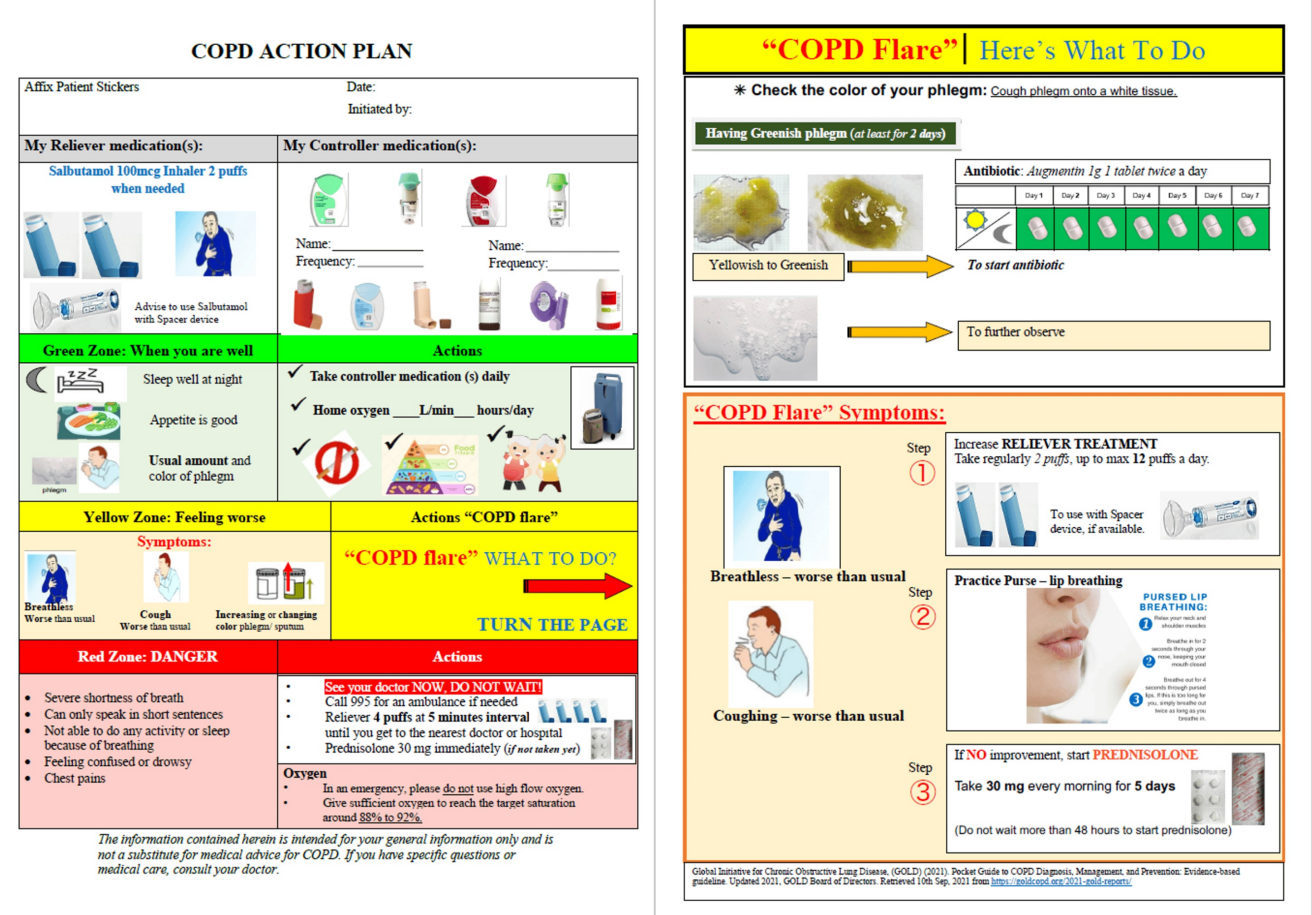
First edition of the pictorial COPD action plan Image credit: The authors COPD: chronic obstructive pulmonary disease

**Figure 7:**
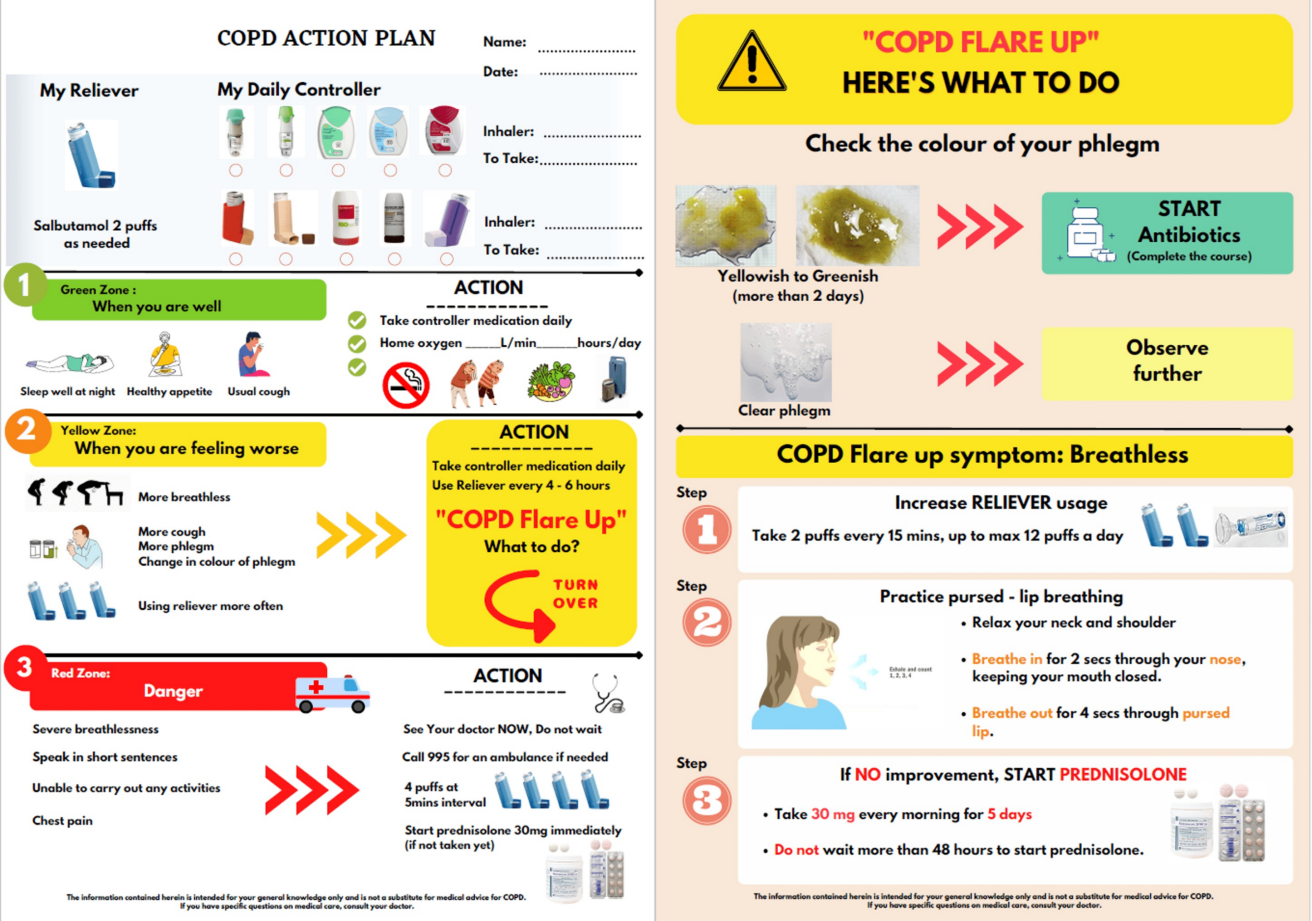
Revised edition of pictorial COPD action plan Image credit: The authors COPD: chronic obstructive pulmonary disease

During the first PDSA cycle (Figure 5), we minimized the clustering of information by removing medical jargon. We also inserted relevant graphics (Figure 6). Afterward, the QI team also conducted an internal review within the respiratory department and the various stakeholders to evaluate the comprehensibility of the action plan. The feedback we received was that the action plan was still too wordy and important points were not emphasized. To address these concerns, we rearranged information in the action plan and important details were highlighted in bold (Figure 7).

**Figure 8:**
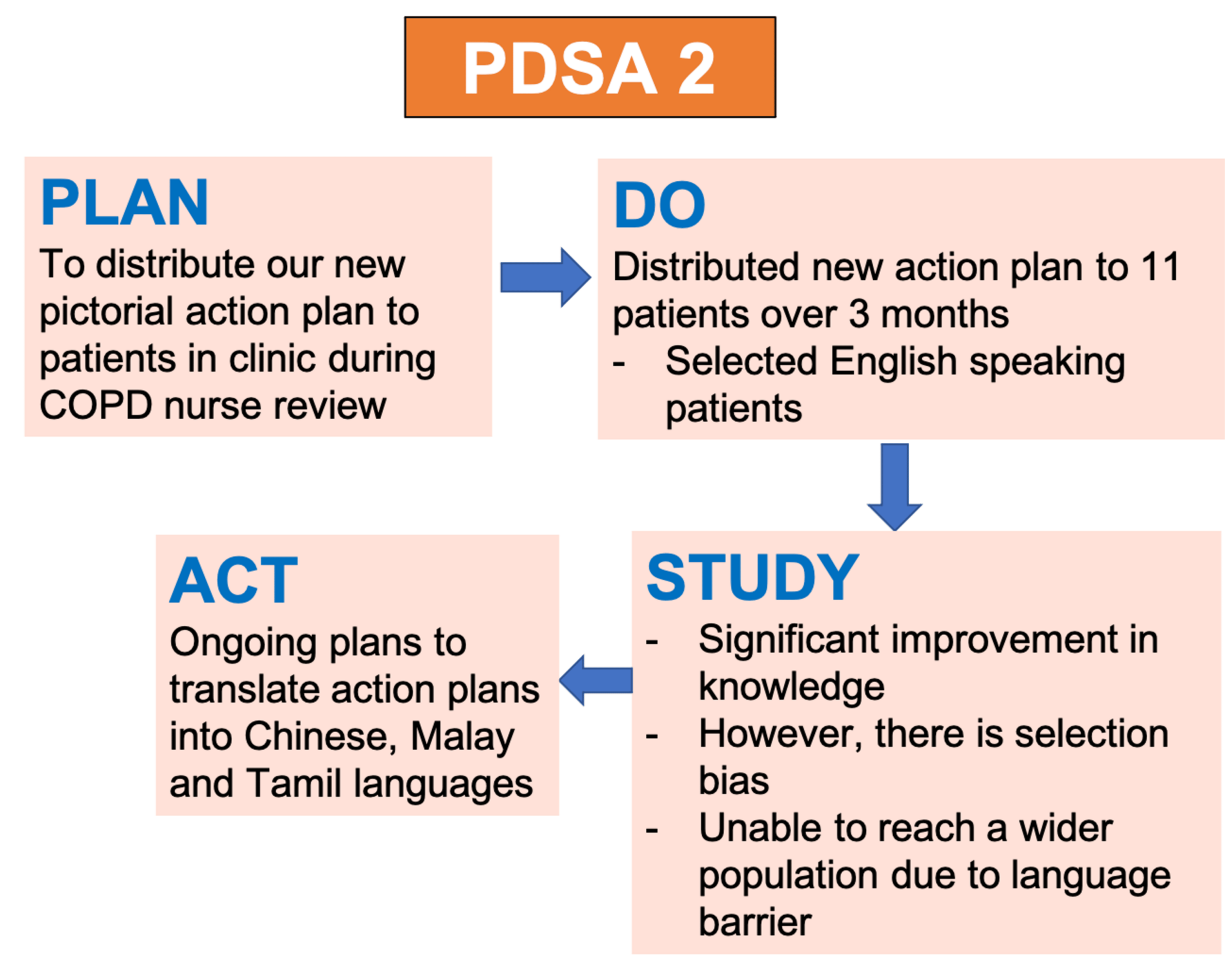
PDSA cycle 2 Image credit: The authors PDSA: Plan-Do-Study-Act

During the second PDSA cycle (Figure 8), we managed to distribute our revised pictorial COPD action plan to 11 English-speaking COPD patients who came to our outpatient respiratory clinic. This was carried out across three months from March to May 2022. As mentioned previously, we would conduct a competency check (Figure 4) after explaining the contents of the action plan to evaluate how much our patients have understood and retained the information. We then compared the knowledge scores we obtained from patients before and after the pictorial action plan was implemented (more details in the "Results" section). During the "study" phase of our PDSA cycle, the QI team recognized that even though there was a significant improvement in knowledge scores, we only targeted English-speaking patients. We were unable to reach a wider audience, as our action plan was only available in English. Hence, there are ongoing plans to translate our action plan into the Chinese, Malay, and Tamil languages.

## Results

Ten English-speaking COPD patients from the outpatient respiratory clinic agreed to be surveyed after administering the original COPD action plan while 11 patients were surveyed after distributing the revised COPD action plan.

With the original COPD action plan, the mean knowledge score obtained from the competency check was 5/10 (n = 10). After receiving the revised plan, the mean knowledge score was 9.64/10 (n = 11). This amounted to an overall improvement of 92.8%, (t (19) = 6.67, p < 0.01, at 95% CI) (1-tailed t-test). Further analysis of the data revealed that the greatest improvement in scores was seen in questions related to recognizing exacerbations and how to manage them.

**Figure 9:**
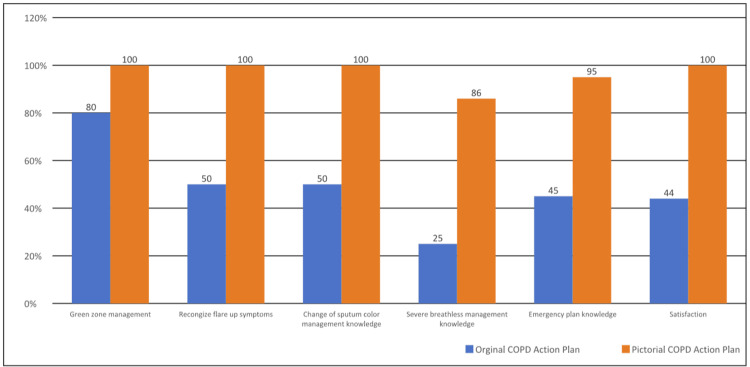
Percentage improvement in knowledge scores before and after the implementation of the pictorial COPD action plan Image credit: The authors COPD: chronic obstructive pulmonary disease

There was a significant improvement in patient satisfaction rates from 44% to 100% (Figure 9). During the 3-month callback, the majority of our patients, 7 out of 11 (63.6%) said they referred back to the new pictorial action plan at home when they were unwell. Out of these seven patients, three of them recognized the need to take a standby course of antibiotics and corticosteroids. These patients improved clinically and did not have to visit the ED to seek medical attention. The remaining patients who did not self-administer treatment or did not refer back to the action plan stated that they had no exacerbations throughout those three months.

## Discussion

COPD action plans have empowered patients to self-manage their conditions. Studies have shown that these action plans improve dyspnoea, decrease respiratory and all cause-related hospitalizations, and increase health-related quality of life [9,10]. Therefore, it is crucial that patients understand the action plan and are willing to use them when necessary.

Pictorially augmented action plans have been used for asthma in various studies [11,12]. They have exhibited benefits in asthma control and health-related quality of life, especially in the illiterate population [13]. A recent study done in Malaysia, which has a similar racial demographic to Singapore, found that pictorial asthma action plans were associated with better asthma control and cost savings in primary care patients [14]. For COPD, there was one small study, which evaluated the comprehensibility of pictorial COPD action plans by employing techniques of guessability and translucency. The study was conducted in a London hospital with 21 adult participants, and the investigators found pictorial action plans to be easily understood [15]. Other than that, our literature review revealed that there was still a lack of similar studies looking at pictorial action plans specifically for COPD patients.

A quality improvement methodology was used to assess the effectiveness of the original COPD action plan in a local context. The problem was evaluated using various QI tools such as the Needs Analysis, Fishbone diagram, Pareto chart, and subsequent Prioritization Matrix. We found a pictorially augmented COPD action plan to be a promising intervention. Upon implementation of our pictorial action plan using PDSA cycles, the data of our QI project showed a statistically significant improvement in knowledge scores between groups who have, and who have not received the new action plan. We achieved our aim of increasing the knowledge scores of our patients during competency checks by 20% over 10 months. A majority of the patients also referred back to our action plan at home.

In the future, we hope to further investigate the clinical impact of a pictorially augmented COPD action plan and to determine if this intervention translates to clinically relevant outcomes such as a decrease in the frequency of ED visits and hospitalization rates.

Limitations

Our study was limited by a small sample size, as it was carried out during the COVID pandemic. There were also potential biases in patient selection and feedback since only English-speaking patients and caregivers were recruited, and the majority of our stakeholders who participated in the Needs Analysis were healthcare professionals. We also recognized that our study had a short follow-up period of three months. Hence, we were unable to draw meaningful conclusions on the long-term impact of our project, specifically with regard to the frequency of COPD exacerbations and healthcare utilization of our patients.

However, we hope that our QI project serves as a primer to future larger-scale studies on pictorial COPD action plans. Furthermore, we expect that the benefits of these action plans will be further validated in upcoming studies.

Sustainability

Our hospital has replaced the existing COPD action plan with the new pictorial version. This new version has been translated into Mandarin, and we are working on translating it into other languages. To help widen our reach and increase accessibility to our COPD action plan, we have uploaded the soft copy version onto the hospital intranet to allow for easy access by other healthcare professionals. The team is also working on creating an application or QR code link to a webpage for easy reference by patients. We also plan to extend our action plan to other hospitals and the primary healthcare setting.

## Conclusions

Pictorially enhanced COPD action plans have been shown to improve our patients’ COPD self-management knowledge. More studies are needed to assess the clinical relevance of this intervention. In the long run, we hope to empower more patients with the skills to self-manage their COPD condition at home.
